# Association of Mannose-Binding Lectin 2 Gene Polymorphisms with Persistent *Staphylococcus aureus* Bacteremia

**DOI:** 10.1371/journal.pone.0089139

**Published:** 2014-03-04

**Authors:** Yong Pil Chong, Ki-Ho Park, Eun Sil Kim, Mi-Na Kim, Sung-Han Kim, Sang-Oh Lee, Sang-Ho Choi, Jin-Yong Jeong, Jun Hee Woo, Yang Soo Kim

**Affiliations:** 1 Department of Infectious Diseases, Asan Medical Center, University of Ulsan College of Medicine, Seoul, Republic of Korea; 2 Department of Laboratory Medicine, Asan Medical Center, University of Ulsan College of Medicine, Seoul, Republic of Korea; 3 Division of Infectious Diseases, Department of Internal Medicine, Kyung Hee University Hospital, Kyung Hee University School of Medicine, Seoul, Republic of Korea; 4 Center for Antimicrobial Resistance and Microbial Genetics, University of Ulsan College of Medicine, Seoul, Republic of Korea; 5 Asan Institute of Life Sciences, Asan Medical Center, University of Ulsan College of Medicine, Seoul, Republic of Korea; Oxford University, United Kingdom

## Abstract

**Objectives:**

Mannose-binding lectin (MBL) is an important component of innate immunity. Structural and promoter polymorphisms in the *MBL2* gene that are responsible for low MBL levels are associated with susceptibility to infectious diseases. The objective of this study was to investigate the association of serum MBL levels and *MBL2* polymorphisms with persistent *Staphylococcus aureus* bacteremia (SAB) in adult Korean patients.

**Methods:**

We conducted a case-control study nested in a prospective cohort of patients with SAB. The study compared 41 patients with persistent bacteremia (≥7 days) and 46 patients with resolving bacteremia (<3 days). In each subject, we genotyped six single-nucleotide polymorphisms in the promoter region (alleles *H/L*, *X/Y*, and *P/Q*) and exon 1 (alleles *A/B*, *A/C*, and *A/D*) of the *MBL2* gene and measured serum MBL concentrations. We also compared *MBL2* genotypes between SAB patients and healthy people.

**Results:**

Patients with persistent bacteremia were significantly more likely to have low/deficient MBL-producing genotypes and resultant low serum MBL levels, than were patients with resolving bacteremia (*P* = 0.019 and *P* = 0.012, respectively). Independent risk factors for persistent bacteremia were metastatic infection (adjusted odds ratio [aOR], 34.7; 95% confidence interval [CI], 12.83–196.37; *P* = 0.003), methicillin resistance (aOR, 4.10; 95% CI, 3.19–29.57; *P* = 0.025), and low/deficient MBL-producing genotypes (aOR, 7.64; 95% CI, 4.12–63.39; *P* = 0.003). Such genotypes were significantly more common in patients with persistent bacteremia than in healthy people (OR, 2.09; 95% CI, 1.03–4.26; *P* = 0.040).

**Conclusions:**

This is the first demonstration of an association of low MBL levels and *MBL2* polymorphisms responsible for low or deficient MBL levels with persistent SAB. A combination of factors, including clinical and microbiological characteristics and host defense factors such as MBL levels, may together contribute to the development of persistent SAB.

## Introduction

*Staphylococcus aureus* bacteremia is one of the most common serious bacterial infections with high morbidity and mortality. Frequently, *S. aureus* bacteremia persists despite several days of appropriate antibiotic therapy. Persistent bacteremia accounts for 6–38% of *S. aureus* bacteremia episodes and is associated with poor clinical outcomes [1]–[4]. Several clinical and microbiological characteristics such as retention of infected devices, endovascular infection, metastatic infection, methicillin resistance, vancomycin minimal inhibitory concentration (MIC) of 2 mg/L, *agr* dysfunction, and resistance to host defense cationic peptides have been suggested as risk factors for persistent bacteremia [1]–[7]. However, these clinical and microbiological factors were not consistent among studies and could explain only a part of persistent *S. aureus* bacteremia. Because *S. aureus* infection is a consequence of the dynamic interaction between bacteria and host defense, some factors related with host response to *S. aureus* may contribute to persistent bacteremia.

For *S. aureus* to invade the host and establish infection, multiple steps such as inoculation and colonization of tissue surfaces, invasion, evasion of the host response, and metastatic spread are required. After the establishment of *S. aureus* infection, antibiotic therapy plays an important role in the eradication of infection. In some patients with *S. aureus* infection, bacteremia persists despite appropriate antibiotic therapy. Decreased or defective host response to *S. aureus* may lead to persistent bacteremia. However, no study has characterized the host response associated with persistent *S. aureus* bacteremia. In animal and *in vitro* studies, mannose-binding lectin (MBL) has been demonstrated to play an important role in the control of *S. aureus* infection [8]–[11]. Therefore, we hypothesized that *MBL2* gene polymorphisms would be associated with persistence of *S. aureus* bacteremia.

MBL is a circulating C-type lectin that plays an important role in innate immunity as the front-line of the host defense system against microbial infection. MBL selectively recognizes the patterns of glycans displayed on the surfaces of a wide range of microorganisms, and then opsonizes antigens and activates the lectin pathway of the complement system [12], [13]. Complement activation results in further opsonization of microorganisms and induction of inflammatory reactions. Human MBL is encoded by a single gene (*MBL2*) located on chromosome 10q21.1 (*MBL1* is a pseudogene). There are six common single-nucleotide polymorphisms (SNPs) in *MBL2* that have a major effect on MBL protein structure and serum levels. Three of these SNPs located in exon 1 at codons 52 (allele *D*), 54 (allele *B*), and 57 (allele *C*), result in amino acid substitutions that interfere with oligomerization of MBL monomers and reduce serum MBL levels [13], [14]. The wild-type allele of these polymorphisms is designed as *A*. There are also two important SNPs in the promoter region at positions −550 (alleles *H* and *L*) and −221 (alleles *X* and *Y*), and one in the 5′ untranslated region at position +4 (alleles *P* and *Q*); these variants influence the rate of transcription and thereby also affect serum MBL levels [13], [14]. These SNPs at the promoter are in strong linkage disequilibrium with SNPs at exon 1 and give rise to seven common haplotypes (*HYPA*, *LYPA*, *LYQA*, *LXPA*, *LYPB*, *LYQC*, and *HYPD*), which show considerable variation in their frequencies between ethnic groups [15]–[18]. MBL deficiency caused by polymorphisms in the *MBL2* gene is associated with increased risk, severity, and frequency of various infections [12], [13], [19]–[21]. The aim of this study was to investigate whether low MBL-producing *MBL2* genotypes or low MBL levels confer an increased risk for persistent *S. aureus* bacteremia.

## Materials and Methods

### Ethics Statement

The Asan Medical Center Institutional Review Board approved this study (IRB number: 2008-0274), following the ethical guidelines for human genome research. All participants provided their written informed consent to participate in this study. The complete protection of their personal data was guaranteed according to the South Korea's Bioethics and Safety Act.

### Study Population

This case-control study, nested in a prospective cohort of patients with *S. aureus* bacteremia was conducted at the Asan Medical Center, a 2700-bed tertiary referral center that admits patients from all of South Korea. From August 2008 to August 2011, all adult patients with *S. aureus* bacteremia were prospectively enrolled in the cohort and followed over a 12-week period. At our hospital, more than 90% of patients with *S. aureus* bacteremia receive infectious disease consultation, and are routinely recommended to undergo follow-up blood cultures at 2–4 day intervals until negative conversion, echocardiography, adequate infection source control, and monitoring of vancomycin trough concentrations (at day 3 and at 3–4 day intervals thereafter). Patients were excluded if: *(i)* they had polymicrobial bacteremia, or *(ii)* they died or were discharged before positive blood culture results. In this cohort, only patients who consented to blood sampling for determination of *MBL2* genotype and MBL concentration were included in the study population. Case patients were those who had an episode of persistent bacteremia, defined as bacteremia for ≥7 days while they were receiving appropriate antibiotic therapy. Control patients were those who had resolving bacteremia, defined as less than 3 days of bacteremia with documentation of all subsequent blood cultures to be negative after the initial positive blood culture (index blood culture). Patients with resolving bacteremia had to have at least one set of follow-up blood cultures 1–3 days after the index blood culture and to have no further positive blood culture results during antibiotic therapy. The duration of bacteremia was calculated as the number of days between the first and last positive blood cultures. Patients with intermediate duration of bacteremia were excluded from analysis to enable clear distinctions between persistent bacteremia and resolving bacteremia. Ultimately, 41 patients with persistent bacteremia and 46 patients with resolving bacteremia were included in the study. To compare *MBL2* genotypes between patients with *S. aureus* bacteremia or persistent bacteremia and healthy persons, we used the published results of a previous study of *MBL2* genotypes in a healthy Korean population [18].

### Data Collection and Study Definitions

Demographic characteristics, underlying diseases or conditions, severity of underlying disease, severity of bacteremia, site of infection, antibiogram results, and patient management were recorded. The system of McCabe and Jackson was used to classify the severity of the underlying disease [22]. Charlson's comorbidity index was used to provide a composite score of comorbid conditions [23]. The severity of bacteremia at the time of the first positive blood culture was assessed using the Pitt bacteremia score [24]. Bacteremia was classified as hospital-acquired if a positive blood culture was obtained from patients who had been hospitalized for 48 h or longer [25].

### Microbiological Data

All *S. aureus* isolates were identified using standard methods. Methicillin resistance was confirmed by polymerase chain reaction (PCR) detection of the *mecA* gene. Vancomycin MICs of methicillin-resistant *S. aureus* (MRSA) isolates were determined by the vancomycin Etest (AB Biodisk, Piscataway, NJ, USA) on Mueller-Hinton agar according to the manufacturer's instructions.

### *MBL2* Genotyping

Genotyping of the *MBL2* gene polymorphisms was performed by PCR and direct sequencing. Six SNPs in the *MBL2* gene were determined: two located in the promoter region (−550 G/C and −221 C/G), one located in the 5′-untranslational region (+4 C/T), and three located in the coding region (codon 52 CGT/TGT, codon 54 GGC/GAC, and codon 57 GGA/GAA) (Table 1). Genomic DNA of all patients was extracted from whole-blood samples using the G-spin™ DNA extraction kit (iNtRON Biotechnology, Suwon, Korea). Extracted DNA was amplified by PCR using the primers listed in Table 1. Each PCR product was sequenced using the PCR amplification primers on an Applied Biosystems 3130*×l* Genetic Analyzer (Applied Biosystems, Carlsbad, CA, USA).

**Table 1:** PCR primers used in the analysis of the *MBL2* gene polymorphisms.

Polymorphism (alleles)	Primer sequence	Product size (bp)	Reference
Promoter −550 (*H*/*L*)	5′-TTGCCAGTGGTTTTTGACTC-3′	302	Huh *et al.* [33]
	5′-GTATCTGGGCAGCTGATTCC-3′		
Promoter −221 (*X*/*Y*)	5′-CAGACACCTGGGTTTCCACT-3′	316	Lee *et al.* [18]
	5′-GAGCATGCCCTCTGTCCTAC-3′		
5′-UTR +4 (*P*/*Q*)	5′-AGTCACGCAGTGTCACAAGG-3′	386	Lee *et al.* [18]
	5′-AGAACAGCCCAACACGTACC-3′		
Exon 1^a^	5′-CCTTCCCTGAGTTTTCTCAC-3′	298	Gomi *et al.* [21]
	5′-ATCAGTCTCCTCATATCCCC-3′		

a Codons 52, 54, and 57 (*A*/*D*, *A*/*B*, and *A*/*C*).

UTR, untranslated region.

### *MBL2* Genotype Groups

According to previous studies documenting that the serum level of MBL is determined by *MBL2* genotypes, we categorized individuals into the following three groups based on their genotypes: high-producing, low-producing, and deficient [13], [19], [20].

### Serum MBL Concentrations

MBL serum levels were determined by a sandwich enzyme-linked immunosorbent assay (ELISA) using the human MBL Duoset ELISA kit (R&D systems, Minneapolis, MN, USA). Samples were initially diluted 1∶400 and processed in triplicate.

### Statistical Analysis

Comparisons were performed between patients with persistent bacteremia and those with resolving bacteremia. Categorical variables were compared using the χ^2^ test or Fisher's exact test, as appropriate. Continuous variables were compared using the Mann-Whitney U test. Deviations from Hardy-Weinberg equilibrium were tested for each individual SNP using the χ^2^ test. Differences in serum MBL concentrations among the high, low, and deficient MBL-production groups were analyzed using Kruskal-Wallis one-way ANOVA on ranks. To identify independent risk factors for persistent bacteremia, all significant variables in the univariate analysis were included in the multiple logistic regression model. The final model was constructed using the forward stepwise selection procedure, and an internal validation was performed with a bootstrap technique. The model was repeatedly applied to 1000 replicated bootstrap samples. Presented 95% confidence intervals (CIs) of all multivariate estimates were derived from the bootstrap analysis. In addition, comparison of *MBL2* genotype groups between patients with *S. aureus* bacteremia or persistent bacteremia and healthy persons [18] was performed using the χ^2^ test. A two-tailed *P* value less than 0.05 was considered statistically significant. All statistical analyses were performed using the SPSS software, version 18.0 (SPSS Inc., Chicago, IL, USA).

## Results

### Study Population

From August 1, 2008 to August 31, 2011, a total of 706 patients with *S. aureus* bacteremia were enrolled in the prospective cohort. A total of 127 of these patients consented to blood sampling. Baseline characteristics were similar between patients whose blood samples were collected and those whose blood samples were not collected (Table S1), although some sites of infection were more frequent in patients whose blood samples were collected. Among 127 patients from whom blood samples were collected, 41 patients with persistent bacteremia and 46 patients with resolving bacteremia were included in the study. Of these 87 patients, 56 (64.4%) had MRSA bacteremia.

### Clinical and Microbiological Characteristics

Demographic characteristics of 87 patients and clinical characteristics and management of *S. aureus* bacteremia are shown in Table 2. The persistent bacteremia and resolving bacteremia groups were similar in age, underlying conditions, severity of underlying disease, severity of bacteremia, and presence or absence of eradicable foci. Furthermore, there were no significant differences between the two groups in the removal of eradicable foci (100% in persistent versus 95.5% in resolving bacteremia group; *P*>0.999) or time to removal of eradicable foci (median 1 days [interquartile range, IQR, 0–3] versus 2 days [IQR, 1–3]; *P* = 0.793). However, patients with persistent bacteremia were significantly more likely to have a methicillin-resistant isolate, infective endocarditis, and metastatic infection, and less likely to have primary bacteremia than were patients with resolving bacteremia. The proportion of MRSA isolates with vancomycin MIC≤1, 1.5, and 2 µg/mL were, respectively, 48.5%, 30.3%, and 21.2%, in patients with persistent bacteremia, and 30.4%, 47.8%, and 21.7%, in patients with resolving bacteremia. These distributions of vancomycin MICs were not significantly different between the two groups (*P* = 0.381).

**Table 2:** Demographic and clinical characteristics of patients with persistent bacteremia and resolving bacteremia caused by *Staphylococcus aureus*.

Characteristic	Total, n = 87 (%)	Persistent bacteremia, n = 41 (%)	Resolving bacteremia, n = 46 (%)	*P* value
Age, median (IQR)	65 (56–72)	65 (58–69)	63 (55–74)	0.708
Male	58 (66.7)	31 (75.6)	27 (58.7)	0.095
MRSA	56 (64.4)	33 (80.5)	23 (50)	0.003
Hospital-acquired infection	37 (42.5)	19 (46.3)	18 (39.1)	0.497
Underlying disease/condition				
Malignancy	34 (39.1)	18 (43.9)	16 (34.8)	0.509
Diabetes	39 (44.8)	19 (46.3)	20 (43.5)	0.789
Chronic renal failure	11 (12.6)	4 (9.8)	7 (15.2)	0.444
Liver cirrhosis	10 (11.5)	4 (9.8)	6 (13.0)	0.743
Ultimately fatal or rapidly fatal disease	19 (21.8)	8 (19.5)	11 (23.9)	0.620
Charlson comorbidity index, median (IQR)	2 (1–4)	2 (1–3)	2 (1–4)	0.746
Pitt bacteremia score, median (IQR)	1 (0–2)	1 (0–2)	1 (0–2)	0.959
Characteristics of infection^a^				
Metastatic infection	26 (29.9)	23 (56.1)	3 (6.5)	<0.001
CVC-related infection	20 (23.0)	13 (31.7)	7 (15.2)	0.068
Infective endocarditis	7 (8.0)	6 (14.6)	1 (2.2)	0.048
Bone and joint infection	24 (27.6)	15 (36.6)	9 (19.6)	0.076
Skin and soft tissue infection	14 (16.1)	4 (9.8)	10 (21.7)	0.129
Primary bacteremia	7 (8.0)	0	7 (15.2)	0.013
Eradicable focus	38 (43.7)	16 (39.0)	22 (47.8)	0.409
Removal of eradicable focus	37/38 (97.4)	16/16 (100)	21/22 (95.5)	0.999

Except where noted, values in parentheses indicate percentages.

a Complication or principal focus of infection at presentation.

IQR, interquartile range; MRSA, methicillin-resistant *S. aureus*; CVC, central venous catheter.

### Allele and Haplotype Frequencies in *MBL2* Gene

In 41 patients with persistent bacteremia, frequencies of exon 1 variants were 74.4% (61/82 alleles) for *A* and 25.6% (21/82) for *B*, with no *C* and *D* allele identified. The allele frequencies at the promoter −550 site were 51.2% (42/82) for *H* and 48.4% (40/82) for *L*; at the promoter −221 site were 85.4% (70/82) for *Y* and 14.6% (12/82) for *X*; and at the 5′-UTR +4 site, 97.6% (80/82) for *P* and 2.4% (2/82) for *Q*. The frequencies of *HYPA*, *LYPA*, *LYQA*, *LXPA*, and *LYPB* haplotypes were 51.2% (42/82), 6.1% (5/82), 2.4% (2/82), 14.6% (12/82), and 25.6% (21/82), respectively. On the other hand, among 46 patients with resolving bacteremia, frequencies of exon 1 variants were 89.1% (82/92) for *A* and 10.9% (10/92) for *B*, with no *C* and *D* allele identified; the allele frequencies of upstream variants were 55.4% (51/92), 44.6% (41/92), 82.6% (76/92), 17.4% (16/92), 89.1% (82/92), and 10.9% (10/92) for *H*, *L*, *Y*, *X*, *P* and *Q* alleles, respectively; and the frequencies of *HYPA*, *HYPB*, *LYPA*, *LYQA*, *LXPA*, and *LYPB* were 54.3% (50/92), 1.1% (1/92), 6.5% (6/90), 10.9% (10/92), 17.4% (16/92), and 9.8% (9/92), respectively. Allele frequencies in the combined study population were in Hardy-Weinberg equilibrium.

### Association of *MBL2* Genotype Groups and Serum MBL Concentrations with Persistent Bacteremia

Homozygous mutated (*B/B*) and heterozygous (*A/B*) genotypes of the coding region were significantly more common in patients with persistent bacteremia than in patients with resolving bacteremia (*P* = 0.016) (Table 3). Patients with persistent bacteremia were significantly more likely than patients with resolving bacteremia to have low or deficient MBL-producing genotypes (*P* = 0.012).

**Table 3:** Comparison of *MBL2* genotypes and serum MBL levels in patients with persistent bacteremia and resolving bacteremia caused by *Staphylococcus aureus*.

Characteristic	Total, n = 87 (%)	Persistent bacteremia, n = 41 (%)	Resolving bacteremia, n = 46 (%)	*P* value
Coding genotype of *MBL2*				0.016
*A*/*A*	57 (65.5)	21 (51.2)	36 (78.3)	
*A*/*B*	29 (33.3)	19 (46.3)	10 (21.7)	
*B*/*B*	1 (1.1)	1 (2.4)	0	
*MBL2* genotype group^a^				0.019
high	53 (60.9)	19 (46.3)	34 (73.9)	
low	29 (33.3)	18 (43.9)	11 (23.9)	
deficient	5 (5.7)	4 (9.8)	1 (2.2)	
Serum MBL, ng/mL, median (IQR)	1389 (668–1882)	1091 (435–1583)	1641 (813–2129)	0.012

a High MBL-producing genotypes: *HYPA/HYPA*, *HYPA/LXPA*, *HYPA/LYPA*, *HYPA/LYQA*, *LYPA/LXPA*, *LYPA/LYQA*, *LYQA/LXPA*; low MBL-producing genotypes: *HYPA/LYPB*, *HYPA/HYPB*, *LXPA/LXPA*, *LXPA/LYPB*, *LYPA/LYPB*; deficient MBL-producing genotypes: *LXPA/LYPB*, *LYPB/LYPB*.

IQR, interquartile range.

Serum samples were drawn at a median 10 days (IQR, 7–16 days) after onset of bacteremia in all study patients. Median sample timing was 9 days (IQR, 8–16 days) in patients with persistent bacteremia and 10 days (IQR, 7–14 days) in patients with resolving bacteremia (*P* = 0.887). Median serum MBL concentration was 1773 ng/mL (IQR, 1428–2143 ng/mL) in the high MBL-producing genotypes group, 686 ng/mL (IQR, 363–890 ng/mL) in the low producing group, and 286 ng/mL (IQR, 223–316 ng/mL) in the MBL-deficient genotypes group. There were significant differences in serum MBL levels among these three genotype groups (*P*<0.001), reflecting the correlation of MBL levels and *MBL2* genotype groups. In agreement with the difference in the proportions of *MBL2* genotype groups between patients with persistent and resolving bacteremia, serum MBL concentrations were significantly lower in patients with persistent bacteremia (*P* = 0.012) (Table 3).

### Multivariate Analysis of Clinical Factors and *MBL2* Genotype Groups

To define independent associations between persistent bacteremia and the significant clinical factors and *MBL2* genotype groups, we constructed a multivariate logistic regression model using bootstrapping. Independent risk factors for persistent bacteremia were metastatic infection (adjusted odds ratio [aOR], 34.7; 95% CI, 12.83–196.37; *P* = 0.003), methicillin resistance (aOR, 4.10; 95% CI, 3.19–29.57; *P* = 0.025), and low/deficient MBL-producing genotypes (aOR, 7.64; 95% CI, 4.12–63.39; *P* = 0.003).

### Comparison of *MBL2* Genotype Groups between Patients with Persistent Bacteremia and Healthy Persons

There was no significant difference in the proportion of low/deficient MBL-producing genotypes between all patients with *S. aureus* bacteremia and healthy persons (39.1% versus 35.7%; *P* = 0.610) (Table 4). Furthermore, there was no significant difference in the proportions of *MBL2* genotype groups between patients with resolving bacteremia and healthy persons. However, low/deficient MBL-producing genotypes were significantly more common in patients with persistent bacteremia than in healthy persons (OR, 2.09; 95% CI, 1.03–4.26; *P* = 0.040).

**Table 4:** Comparison of *MBL2* genotype groups in patients with *Staphylococcus aureus* bacteremia or patients with persistent bacteremia versus healthy people.

Genotype group^a^	*Staphylococcus aureus* bacteremia						Healthy control (n = 129) (%)
	Overall (n = 87) (%)	OR (95% CI)^b^	*P* b	Persistent bacteremia (n = 41) (%)	OR (95% CI)^c^	*P* c	
High	53 (60.9)	1.00 (Reference)	…	19 (46.3)	1.00 (Reference)	…	83 (64.3)
Low or deficient	34 (39.1)	1.16 (0.66–2.03)	0.610	22 (53.7)	2.09 (1.03–4.26)	0.040	46 (35.7)

a High MBL-producing genotypes: *HYPA/HYPA*, *HYPA/HYQA*, *HYPA/LXPA*, *HYPA/LYPA*, *HYPA/LYQA*, *LYPA/LXPA*, *LYPA/LYPA*, *LYPA/LYQA*, *LYQA/LXPA*, *LYQA/LYQA*; low MBL-producing genotypes: *HYPA/LYPB*, *HYPA/HYPB*, *LXPA/LXPA*, *LXPA/LYPB*, *LYPA/LYPB*, *LYQA/LYPB*; deficient MBL-producing genotypes: *LXPA/LYPB*, *LYPB/LYPB*.

b Patients with *S. aureus* bacteremia versus healthy control subjects [18].

c Patients with persistent *S. aureus* bacteremia versus healthy control subjects.

## Discussion

We investigated the associations between *MBL2* gene polymorphisms, MBL levels, and persistent *S. aureus* bacteremia and demonstrated that low/deficient MBL producing-genotypes and low MBL levels are significant risk factors for persistent bacteremia. Persistent bacteremia that fails to respond to appropriate antibiotic therapy is frequently encountered in the management of patients with *S. aureus* bacteremia and is associated with poor clinical outcomes [1]–[4]. Many recent studies have defined ‘persistence’ as *S. aureus* bacteremia lasting for ≥7 days despite appropriate antibiotic therapy [1], [2], [5], [26]. Various risk factors may contribute to the development of persistent bacteremia: (i) clinical factors such as infection site, delayed removal of infection source, and metastatic infection [1], [2], [6]; (ii) microbiological characteristics of *S. aureus* such as methicillin resistance, *agr* dysfunction, resistance to host defense cationic peptides, and higher vancomycin MICs [4]–[7]; (iii) pharmacokinetic and pharmacodynamic characteristics of the antibiotic such as vancomycin trough level and the ratio of the area under the curve to the MIC [6], [27]; (iv) factors associated with host defense [28]. It seems highly likely that a combination of these risk factors, rather than a single risk factor, causes persistent bacteremia. In this study, patients with persistent bacteremia were more likely than patients with resolving bacteremia to have CVC-related infection, bone and joint infection, MRSA infection, infective endocarditis, and metastatic infection, consistent with the findings of previous studies [1], [2], [6]. In addition, patients with persistent bacteremia were more likely to have low/deficient MBL-producing genotypes and consequently lower serum MBL levels, than were patients with resolving bacteremia. Even after multivariate analysis adjusting for other clinical variables, low/deficient MBL-producing genotypes were independent risk factors for persistent bacteremia. Therefore, *MBL2* genotypes could be of clinical interest as a molecular marker to identify patients with *S. aureus* bacteremia who are at risk for persistent bacteremia. To our knowledge, this is the first study of *MBL2* genotyping and MBL levels that focused on patients with persistent *S. aureus* bacteremia.

There are no data regarding host defense factors associated with persistent *S. aureus* bacteremia. However, one animal study evaluated the immune response associated with persistent *S. aureus* infection. Using an experimental mouse model of persistent *S. aureus* infection, Ziegler et al. proposed that T cell hyporesponsiveness to *S. aureus* antigens may contribute to the failure of the host immune system to control the infection, resulting in persistent infection [28]. This finding should be evaluated in the context of persistent *S. aureus* bacteremia in humans.

Innate immunity plays an essential role in alerting the host immune system to the presence of pathogens, as well as their clearance. Therefore, a defect or alteration in one of the components of the innate immune system could contribute to development of persistent *S. aureus* bacteremia. MBL plays an important role in innate immunity as a pattern recognition molecule, which activates the complement system and mediates phagocytosis after binding to specific carbohydrates on the surfaces of several types of bacteria, fungi, and viruses [11]–[13]. Serum MBL levels are mainly determined by SNPs in the promoter and exon 1 of the *MBL2* gene [13], [20]. Genotypes associated with low serum MBL levels have been correlated with an increased risk, severity, and frequency of bacterial infections (especially, *Neisseria meningitidis*, *Streptococcus pneumoniae*), as well as fungal and viral infections [12], [13], [19]–[21], [29]. MBL strongly binds to *S. aureus* and enhances complement activation by the lectin pathway, opsonophagocytosis, and induction of proinflammatory responses [8], [9], [11], [30]. In a study using *MBL* knockout mice, Shi et al. found that all *MBL*-null mice died 2 days after intravenous inoculation of *S. aureus*, whereas only 45% of wild-type mice died, indicating that MBL plays a key role in limiting the complications associated with *S. aureus* infection [10]. However, despite this evidence, there are few clinical data regarding the association of low or deficient MBL levels with *S. aureus* infection in humans. Kars et al. showed that MBL deficiency is associated with recurrent staphylococcal disease presenting as furunculosis or carbuncles in 28 members of one particular family with a high prevalence of *S. aureus* furunculosis [29]. In the present study, the proportion of low/deficient MBL-producing genotypes between patients with *S. aureus* bacteremia and healthy persons was similar. However, low/deficient MBL-producing genotypes were significantly more common in patients with persistent *S. aureus* bacteremia than in healthy persons or in patients with resolving bacteremia. This would mean that MBL might play a more important role in the clearance of *S. aureus* bacteremia than in initial protection against *S. aureus* infection.

Our study has several limitations. Among patients with *S. aureus* bacteremia, only about 20% of patients consented to blood sampling for determination of *MBL2* genotype and serum MBL level. The proportion of patients with persistent bacteremia was much higher in the study population than in the entire cohort [6], probably because patients with persistent bacteremia were more willing to consent to blood sampling than patients with resolving bacteremia. Therefore, selection bias might be present. However, because baseline characteristics were similar between patients from whom blood samples were or were not collected, and risk factors for persistent bacteremia were similar to those in previous studies, it is reasonable to consider our study population as a random sample from all patients with *S. aureus* bacteremia. To verify the association with low MBL levels and persistent bacteremia, a further study should be performed with large numbers of patients that accurately reflect the cohort of patients with *S. aureus* bacteremia. In addition, our data regarding *MBL2* genotypes of healthy controls were borrowed from a previous study conducted in Korea [18]. Thus, we could not directly compare serum MBL levels between the study groups and healthy controls. Because the distributions of alleles and haplotypes in healthy persons in that study [18] were very similar with those observed in other studies in Korea [31], [32], it is likely that the *MBL2* genotypes of healthy controls used in our study accurately reflect those of the general Korean population.

## Conclusions

This study is the first to investigate the association of MBL levels and *MBL2* polymorphisms with persistent *S. aureus* bacteremia, and the results suggest that patients with *S. aureus* bacteremia with low/deficient MBL producing *MBL2* genotypes have a higher risk for developing persistent bacteremia. We conclude that a combination of factors, including clinical characteristics of patients, microbiological characteristics of *S. aureus* strain, and host defense factors such as MBL levels, may together contribute to the development of persistent *S. aureus* bacteremia.

## Supporting Information

Table S1 **Demographic and clinical characteristics of patients whose blood samples were or were not collected.**
(DOCX)
