# Supplementary material for: Association of Mannose-Binding Lectin 2 Gene Polymorphisms with Persistent Staphylococcus aureus Bacteremia
Source: PLoS One. 2014 Mar 4;9(3):e89139. doi: 10.1371/journal.pone.0089139 (PMC3942407; doi:10.1371/journal.pone.0089139)
Supplement: Table S1 — Demographic and clinical characteristics of patients whose blood samples were or were not collected. (DOCX) [file pone.0089139.s001.docx]

Table S1. Demographic and clinical characteristics of patients whose blood samples were or were not collected

| Characteristic | Collected,  n = 127 (%) | Not collected,  n = 579 (%) | *P* value |
| --- | --- | --- | --- |
| Age, median (IQR) | 63 (53–71) | 63 (52–71) | 0.786 |
| Male | 83 (65.4) | 372 (64.2) | 0.814 |
| MRSA | 72 (56.7) | 343 (59.2) | 0.597 |
| Hospital-acquired infection | 53 (41.7) | 386 (66.7) | 0.001 |
| Underlying disease/condition |  |  |  |
| Solid tumor | 41 (32.3) | 227 (39.2) | 0.145 |
| Diabetes | 45 (35.4) | 180 (31.1) | 0.341 |
| Chronic renal failure | 15 (11.8) | 87 (15.0) | 0.351 |
| Liver cirrhosis | 16 (12.6) | 91 (15.7) | 0.375 |
| Pitt bacteremia score, median (IQR) | 1 (0–2) | 1 (0–2) | 0.156 |
| Site of infection^a^ |  |  |  |
| CVC-related infection | 27 (21.3) | 218 (37.7) | <0.001 |
| Infective endocarditis | 10 (7.9) | 18 (3.1) | 0.013 |
| Bone and joint infection | 29 (22.8) | 36 (6.2) | <0.001 |
| Skin and soft tissue infection | 22 (17.3) | 48 (8.3) | 0.002 |
| Postoperative wound infection | 11 (8.7) | 40 (6.9) | 0.490 |
| Primary bacteremia | 18 (14.2) | 84 (14.5) | 0.999 |

Except where noted, values in parentheses indicate percentages.

^a^ Principal focus of infection at presentation.

IQR, interquartile range; MRSA, methicillin-resistant *Staphylococcus aureus*; CVC, central venous catheter.
